# Automated and robust beam data validation of a preconfigured ring gantry linear accelerator using a 1D tank with synchronized beam delivery and couch motions

**DOI:** 10.1002/acm2.12946

**Published:** 2020-07-02

**Authors:** Nels C. Knutson, Matthew C. Schmidt, Francisco J. Reynoso, Yao Hao, Thomas R. Mazur, Eric Laugeman, Geoffrey Hugo, Sasa Mutic, H. Harold Li, Wilfred Ngwa, Bin Cai, Erno Sajo

**Affiliations:** ^1^ Department of Radiation Oncology Washington University School of Medicine St. Louis MO USA; ^2^ Department of Physics University of Massachusetts Lowell Lowell MA USA

**Keywords:** automated commissioning, quality assurance, TPS validation

## Abstract

**Purpose:**

To develop an efficient and automated methodology for beam data validation for a preconfigured ring gantry linear accelerator using scripting and a one‐dimensional (1D) tank with automated couch motions.

**Materials and methods:**

Using an application programming interface, a program was developed to allow the user to choose a set of beam data to validate with measurement. Once selected the program generates a set of instructions for radiation delivery with synchronized couch motions for the linear accelerator in the form of an extensible markup language (XML) file to be delivered on the ring gantry linear accelerator. The user then delivers these beams while measuring with the 1D tank and data logging electrometer. The program also automatically calculates this set of beams on the measurement geometry within the treatment planning system (TPS) and extracts the corresponding calculated dosimetric data for comparison to measurement. Once completed the program then returns a comparison of the measurement to the predicted result from the TPS to the user and prints a report. In this work lateral, longitudinal, and diagonal profiles were taken for fields sizes of 6 × 6, 8 × 8, 10 × 10, 20 × 20, and 28 × 28 cm^2^ at depths of 1.3, 5, 10, 20, and 30 cm. Depth dose profiles were taken for all field sizes.

**Results:**

Using this methodology, the TPS was validated to agree with measurement. All compared points yielded a gamma value less than 1 for a 1.5%/1.5 mm criteria (100% passing rate). Off axis profiles had >98.5% of data points producing a gamma value <1 with a 1%/1 mm criteria. All depth profiles produced 100% of data points with a gamma value <1 with a 1%/1 mm criteria. All data points measured were within 1.5% or 2 mm distance to agreement.

**Conclusions:**

This methodology allows for an increase in automation in the beam data validation process. Leveraging the application program interface allows the user to use a single system to create the measurement files, predict the result, and then compare to actual measurement increasing efficiency and reducing the chance for user input errors.

## 1 INTRODUCTION

Recently, a new‐generation ring gantry linear accelerator (linac), Halcyon (Varian Medical System, Palo Alto, CA), was released to help address the global need of image‐guided radiotherapy (IGRT). This linear accelerator can deliver 6‐MV flattening‐filter‐free beams with rapid gantry rotation up to four rotation per minute (RPM) with a compact jawless treatment head equipped with double stack multileaf collimators (MLCs). This linear accelerator comes with a preconfigured representation within the treatment planning systems (TPS) but must be validated by the user. General guidelines to the overall treatment accuracy achievable within radiation oncology (~5%) have been published in the literature.^1^ The general quality assurance guidelines for radiation oncology have been well described in the literature.^2^, ^3^, ^4^, ^5^ In particular, the quality assurance needed for validation of the TPS is well described.^6^, ^7^, ^8^, ^9^, ^10^ Recent publications have applied the methodologies to the Halcyon platform.^11^, ^12^, ^13^ Others have looked at validating the automated daily quality assurance (QA) which is currently available for Halcyon and TrueBeam platforms.^14^ This work looks to extend this automated approach to the beam data validation that is required upon initial installation of the Halcyon.

Treatment planning systems validation is an important and critical component of the commissioning process in radiation therapy. The accuracy of the dose calculation in a water phantom form a reference QA test set of fields should be within 2% dose difference or 2 mm distance to agreement according to the published quality assurance task group report 40^4^ and within 2% in the high‐dose area and within 3 mm of the penumbra according to published practice guide lines.^8^ The importance of these guidelines is highlighted by the fact that there is variability in the accuracy of commissioning as reported by third party auditing done in the United States.^15^, ^16^, ^17^, ^18^, ^19^ Of interest, as reported by Molineu et al, the incidents of failing to meet the third party audit were reduced in preconfigured TPS and delivery machines combinations.^17^ The fact that a preconfigured TPS and linear accelerator combination is attractive due to the reduction in user inputs does not mean the user does not have to complete TPS validation.

Currently, TPS validation is generally done after inputting commissioning data from a three‐dimensional (3D) scanning tank. For the Halcyon, the TPS model comes preconfigured and does not take any user‐defined TPS data input. The physicist must validate this preconfigured beam data. Comparison to 3D tank water scans are the gold standard for this validation, however, alternative methods have been suggested in the literature.^20^, ^21^, ^22^, ^23^, ^24^ Of concern with the Halcyon is the bore size (100 cm diameter), which limits clearance when using a traditional 3D tank size (~70 × 70 × 56 cm). The potential sag associated with supporting the weight of a full 3D tank (~270 kg) on the couch could also make leveling difficult. Furthermore, the associated cost of the 3D tank systems can be burdensome in a resource limited environment.

Using a one‐dimensional (1D) tank with synchronized couch motions with beam delivery could allow the user to validate the TPS in a manner similar to traditional 3D tank methods, however, with less equipment and storage space needed. The use of the extensible markup language (XML), allows the user to program couch motions as a function of monitor units delivered.^25^ Cross‐beam profiles can be reconstructed by moving the couch along the beam while recording the current in field and reference ionization chambers. In the TPS, plans with multiple isocenter locations along the axis of the profile desired by the user are calculated to simulate the tank movement relative to isocenter during the measurement. Doses to central voxels at different depths for each plan represent different detector positions would be analogous to extracting profiles at different depths, similar to conventional 3D tank data. Leveraging scripting, this can be done programmatically such that the user is defining what to validate, and then the program not only extracts the TPS data to be validated, but also writes the XML file that will be used to measure the data. The measured data can then be fed back into the program for analysis allowing the user a feedback system to determine if the beam data validation is complete or needs further review. This feedback loop connecting the measurement on the machine to the TPS calculation could provide an efficient and robust beam data validation methodology not seen in previous works.

## 2 MATERIALS AND METHODS

### 2.A A robust workflow with feedback

**Fig. 1:**
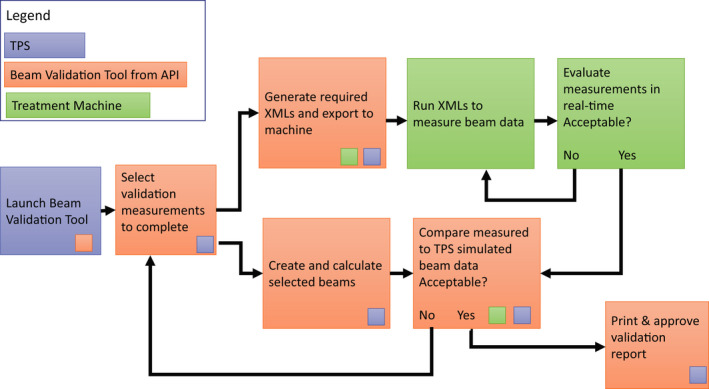
Flowchart for the beam validation process. Each step taken is color coded indicating what system is being used. The small colored squares indicate a connection point to the treatment planning system (TPS), the application programing interface (API), or the treatment machine within a given step. The TPS, beam validation tool, and treatment machine are connected through two separate feedback loops allowing the user to design a custom validation data set that leverages the API to connect the TPS and treatment machine.

A beam validation script was designed using the TPS (Eclipse v15.6, Varian Medical Systems, Palo Alto CA) application programing interface (API) to allow the user to select the set of beam validation parameters to simulate and then measure. These options included depth profiles, lateral profiles, and diagonal profiles at depths selected by the user. Once the user selects the desired data set for validation the script then designs the XML file to be opened on the machine for the measurements. From the user input, that is, the desired profiles, depths of measurements, and detector size used, the script automatically calculates the measurement geometry within the TPS and extracts the expected measurement result for analysis against the measurement. Once the measurement is completed on the machine, this data is provided as feedback into the software for comparison. The comparison between the measured result and the expected from the TPS was completed both graphically and using a 1D gamma analysis^26^ with user configurable tolerances for dose difference and distance to agreement. The percentage of points within a set of data with a gamma value <1 was then reported for the user. At which point if the user finds the results acceptable, they can export a report or choose to add additional validation tests. Figure 1 shows a flowchart of this workflow with the beam validation tool directly linking the TPS outputs to the measurements on the treatment machine via the application programing interface.

### 2.B Measurement

**Fig. 2:**
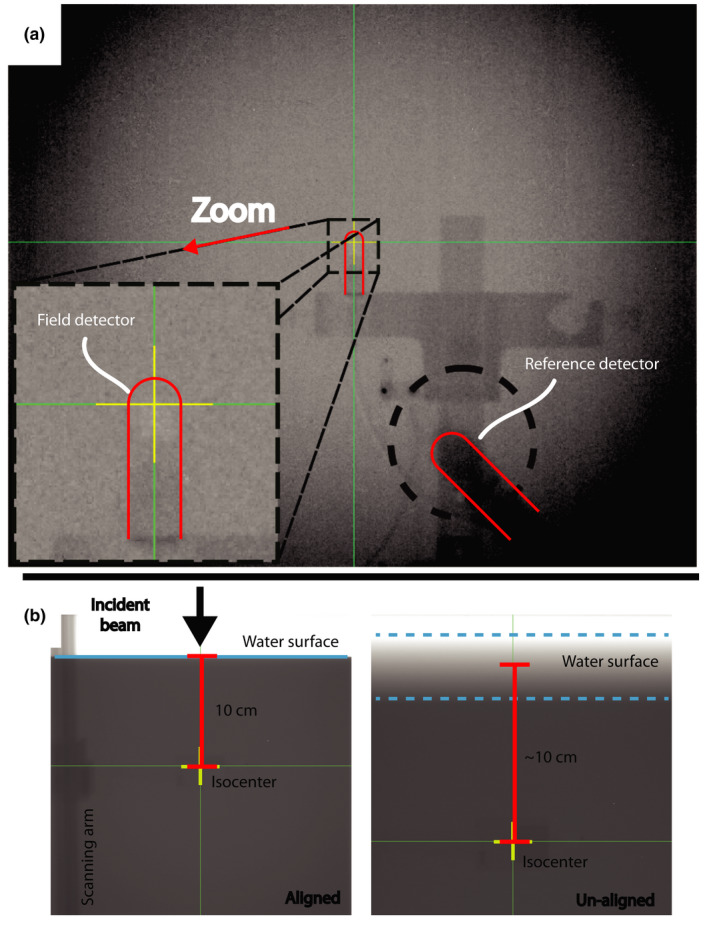
(a) AP MV image that is used to confirm the alignment of the field detector with isocenter and the placement of the reference detector in the corner of the field for a crossline scan. (b), Two MV images that is used to set the SSD to 90 cm. The image on the left was taken at 84.3° allowing the user to identify the water surface. The image on the right was taken at 90 degrees and the water surface is more difficult to identify.

For this work, an example subset of data was selected for beam validation for the Halcyon. These measurements were completed on the Halcyon by selecting the generated XML and recording the output of a data logging electrometer. This data was readout by an inhouse software and displayed during the measurement in real‐time via a graphical user interface (GUI). The field sizes selected were 6 × 6, 8 × 8, 10 × 10, 20 × 20, and 28 × 28 cm^2^. X and Y profiles were taken at 1.3, 5, 10, 20, and 30 cm depths. Diagonal profiles were completed at the same depths for 28 × 28 cm^2^ field size. For alignment, the tank arm was leveled with a spirit level, the tank was initially aligned using the lasers outside the bore to set the detector at laser isocenter and the source to surface distance of the water. Once the initial setup was completed the treatment couch was used to position the tank at the treatment isocenter. The reference detector was taped into place on the bore in a position away from the central axis position of the source. All cabling was routed perpendicular to the scan path to try to minimize the amount of cable in the beam. Once setup was complete, it was then confirmed with MV imaging. An AP MV image taken to set the couch lateral and longitudinal positions such that the field detector was at radiation isocenter. The reference detector was confirmed to be in an appropriate position as not to obscure the field detector during the scan [Fig. 2(a)]. The source to surface distance (SSD) was set using MV imaging through the side of the tank and measuring from isocenter to the water surface [Fig. 2(b)]. To ensure a crisp image of the water surface, the imaging was completed at an angle equal to 90° minus the angle between the source and water surface for a given depth of the isocenter. The angle between the water surface and the source was calculated as the arctangent of the depth of the isocenter over 100 cm. For example, at 100 cm SSD, this would correspond to depth of 0 and therefore gantry imaging angle of 90° would be appropriate. For 90 cm SSD, the gantry was set to 84.3° [Fig. 2(b) on the left] producing a crisp image of the water surface vs an image at 90° in which the water surface is hard to identify [Fig. 2(b) on the right].

All measurements were completed using a 0.13 cc field scanning chamber with a 0.13 cc reference chamber (CC13, IBA Dosimetry, Schwarzenbruck, Germany). A 38 × 41 × 37 cm^3^ 1D tank (1D Scanner, Sun Nuclear Corporation., Melbourne, FL, USA) was used for the measurements. All measurements were completed at 90 cm SSD. All measurements were completed using continuous scans. For the lateral profile scans, the sampling rate of the electrometer was 300 ms for all scans. All crossline (across the bore, X axis) and inline (along the bore, Y axis) scans were completed at 1.5 mm per second scan speed. All diagonal scans were completed with an X and Y axis scan speed of 2 mm per second each; therefore, a speed of 2.83 mm per second along the diagonal axis. For depth scans, the field chamber scanned from 30 cm depth to the surface at 2.5 mm/s with a 100 ms electrometer sampling rate. All scans were completed accounting for the effective point of measurement.

**Fig. 3:**
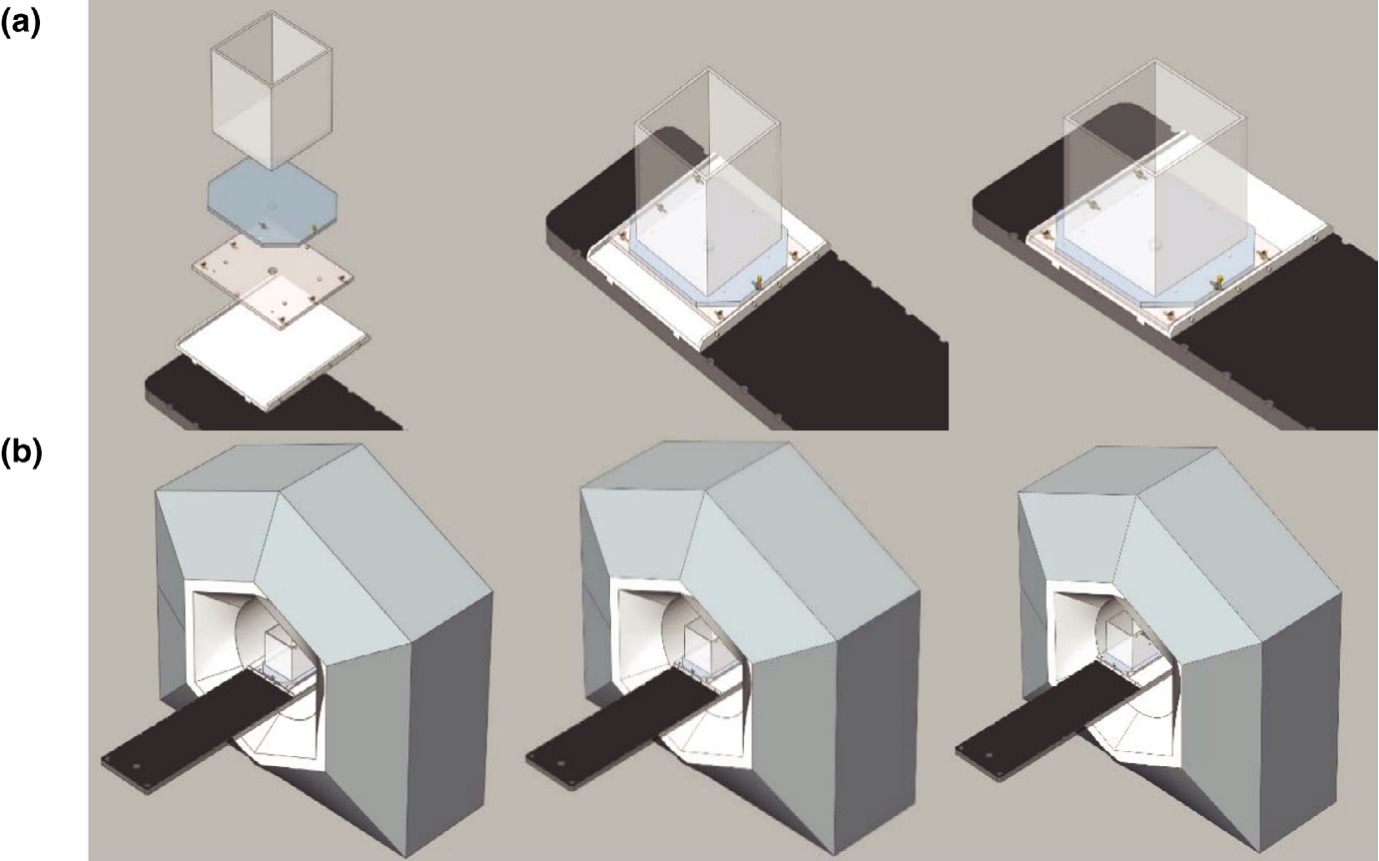
Computer aided design drawing of the sliding platform. (a) The entire platform assembly along with the exploded view of all the constituent parts (the base plate (white), which indexes to the couch (black), the middle plate (tan) with rollers (yellow) allowing for lateral offset, and finally the tank (gray) rests on top. (b) Shows the tank going from the standard position with the couch centered, then the tank platform in the laterally offset position, and finally both the tank and couch in the laterally offset position.

The ring gantry linear accelerator caused the scan range to be limited in the lateral direction by the bore to approximately 20 cm and limits the achievable scan length of crossline and diagonal profiles. Therefore, a sliding platform was designed to allow the user to take scans at an offset position across the scan range and combine multiple scans at different tank positions, thus extending the scan range. Images from the computer‐aided design (CAD) model are shown in Fig. 3. The platform consists of a base plate that indexes to the treatment couch, a second plate with rollers that can be locked into the base plate, and a top plate that has a peg that fits into the middle plate that allows the top platform to rotate. One can perform a scan with the tank offset 9.5 cm laterally from isocenter. With the range of 20 cm, one can effectively scan from +19.5 cm to −0.5 cm in a single scan and then repeat it with the opposite offset to achieve a scan from +0.5 cm to −19.5 cm. The two scans can be combined using the 1 cm of overlap. The same principle was used for diagonal scans. The tank was offset laterally via the mechanical platform. Since there is no limitation concern in the longitudinal direction, there is no need for a longitudinal offset if the lateral and longitudinal coordinates match. Imaging was repeated to ensure the offset was applied accurately. Repeating imaging also allowed the water surface additional time to settle.

### 2.C TPS simulation

To simulate off axis scanning with the 1D tank and automated couch movements, the 1D tank phantom was created within the TPS as a unit density phantom image set of the same dimensions as the physical 1D tank. To simulate a scan, the script created a course per field size. Within a course, a plan was created for each couch position used for a scan. The dose to a 0.5 × 0.5 × 0.5 cm^3^ reference voxel simulating the 0.13‐cm^3^ field ionization chamber at a given depth was recorded for a fixed number of monitor units. The aggregate of all these calculations of dose to the reference voxel for all couch positions was used to construct the TPS output for comparison to measurements. Since the TPS assumes radial symmetry in the beam, half profiles were simulated and mirrored for comparison to measurement. All profiles were divided into three regions; in‐field, the penumbra, and the umbra. The data point spacing was varied based on the region. A 5 mm spacing was used in‐field and outside of the field (the umbra). One millimeter spacing was used in the penumbra and in the transition between the penumbra and the in‐field region and in the transition between the penumbra and the umbra regions. For central axis depth scans, the dose to the central pixels for depths ranging from 0 to 30 cm with 5 mm spacing were extracted for the plan with isocenter in the center of tank representing X = Y = 0. This was completed for each field size.

The simulations were completed for 6 × 6, 8 × 8, 10 × 10, 20 × 20, and 28 × 28 cm^2^ fields for inline and crossline profiles at 1.3, 5, 10, 20, and 30 cm depths. Diagonal profiles were completed for the 28 × 28 cm^2^ field at the same depths by varying the x and y isocenter coordinates and converting this to radial position by adding them in quadrature. Once collected, this data was converted to the standard format and saved for comparison to the measurement input by the user.

### 2.D Beam data validation comparisons

Once given the user input measurement data, the 1D gamma profiles were calculated for all pairs of depth and off axis profiles (measured vs TPS expected). The dose difference and distance to agreement criteria were varied to investigate the agreement between the data sets, that is, the percentage of points with a gamma value < 1. The only post processing used on the data was to center the lateral profiles,normalize to the average of the three center dose values for the lateral profiles, and to normalize to the maximum dose values for the depth profiles. The dose differences and distance to agreement distributions were also calculated individually as well. The results were then output for user review and approval.

## 3 RESULTS

### 3.A Depth profiles

**Fig. 4:**
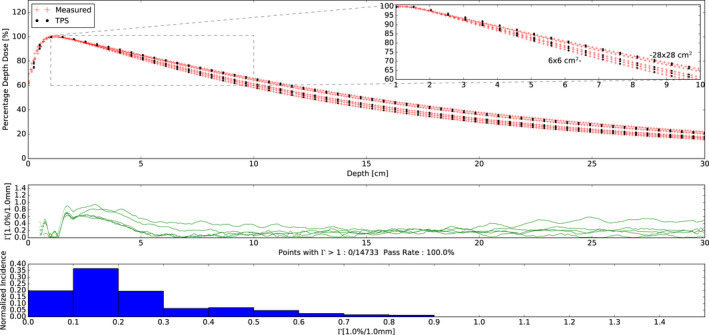
(Top) Percentage depth dose measurements and treatment planning system (TPS) simulations for 6 × 6, 8 × 8, 10 × 10, 20 × 20, and 28 × 28 cm^2^ field sizes. The zoomed insert shows depths from 1 to 10 cm depth. All profiles were normalized to their own max dose. Note the monotonic increase in depth dose with field size. (Middle) Corresponding gamma values with 1%, 1 mm criteria as a function of depth. All gamma values were less than 1 and are shown in green. (Bottom) Histogram plot of the gamma values.

Comparisons between the measured percentage depth dose profiles and TPS simulated depth dose profiles for all field sizes yielded a gamma value < 1 for 1% and 1 mm criteria (100% passing rate with gamma mean: 0.236, max: 0.943). The measured and TPS simulated percentage depth dose profile data, corresponding gamma values with 1% and 1 mm criteria, and histogram of gamma values are shown in Fig. 4. In general, the agreement was excellent for all depth dose profiles measured being within 1.5% dose difference or 1 mm distance to agreement.

### 3.B Off axis profiles

**Fig. 5:**
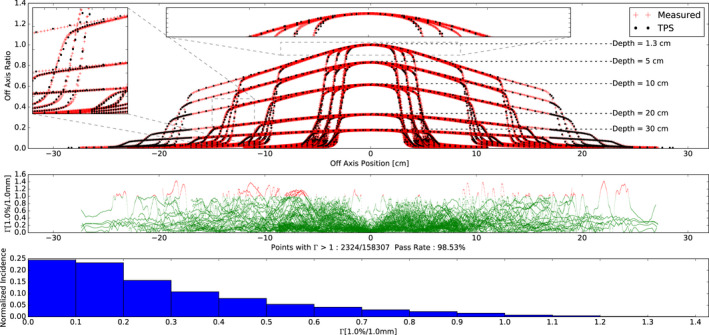
(Top) Off axis profile measurements and TPS simulations for 6 × 6, 8 × 8, 10 × 10, 20 × 20, and 28 × 28 cm^2^ field sizes at depths of 1.3, 5, 10, 20, 30 cm depths. Diagonal profiles are also plotted for the 28x28 cm^2^ field size. For plotting all profiles were normalized to the max dose at 1.3 cm depth. For comparison all profiles were normalized to the central axis. The zoomed insets are provided in a sample penumbra region and infield. (Middle) Corresponding gamma values with 1%, 1 mm criteria as a function of off axis. Gamma values > 1 are shown in red while gamma values < 1 are shown in green. (Bottom) Histogram plot of the gamma values.

All comparisons between measured profiles and TPS simulated profiles yielded a gamma value < 1 for 1.5% and 1.5 mm criteria (100% passing rate with gamma mean: 0.185, max: 0.954) (data not shown). The comparison with a gamma criterion of 1% and 1 mm yielded 98.5% of points with a gamma value < 1 (mean gamma: 0.289, max gamma: 1.430). The measured and TPS simulated profile data, corresponding gamma values (1%, 1 mm), and histogram of gamma values are shown in Fig. 5. The areas that exceed a gamma value of 1 are shown in red and tend to be in the beginning and/or end of the penumbra of each field. However, in general, the agreement was excellent for all profiles measured, with all data being within 1.5% dose difference or 2 mm distance to agreement.

## 4 DISCUSSION

In this work, the preconfigured ring gantry system described met the Task Group 40^4^ and AAPM MPPG5.a^8^ guidelines with all measured doses being within 1.5% or 2 mm of the TPS calculation, giving the user confidence in the accuracy of the TPS dose calculation algorithm. The setup of the system took approximately 30 min. The script to generate the calculations and XML files took approximately 15 min to complete and was ran during the setup of the water tank. Each depth dose scan took 2 min. Each lateral scan took between 1.5 min and approximately 3 min depending on the field size. A buffer of at least 1 min between scans was used to allow for potential water motion to settle. This buffer was increased by 2 min for all scans that required a tank shift and each half scan was considered as a scan. The take down of the system took approximately 30 min. All the analysis was generated as the scanning process was completed and a summary was generated during the take down of the equipment. Table 1 summaries the time needed for the 75 completed scans. This took just over 4 hours. This process allows two trained physicists to complete beam data validation (measurement, calculation, and comparison) within a single day.

**Table 1:** Summary of times needed for the completed scans.

Scan type	Scan length (cm)	Scan speed (cm/s)	Time between scans (min)	Field size (cm)	Time per scan (min)	Number of scans	Total time (min)
Depth dose	30	0.25	0	ALL	2.00	5	10.00
Inline	22	0.15	1	6 × 6	2.46	5	12.31
Inline	22	0.15	1	8 × 8	2.46	5	12.31
Inline	29	0.15	1	10 × 10	3.24	5	16.19
Inline	35	0.15	1	20 × 20	3.91	5	19.53
Inline	45	0.15	1	28 × 28	5.02	5	25.08
Crossline	18	0.15	1	6 × 6	2.02	5	10.08
Crossline	18	0.15	1	8 × 8	2.02	5	10.08
Crossline	18	0.15	1	10 × 10	2.02	5	10.08
Crossline	18	0.15	3	20 × 20	2.05	10	20.50
Crossline	18	0.15	3	28 × 28	2.05	10	20.50
Diagonal	25.46	0.28	3	28 × 28	1.55	10	15.50
					Scanning totals	75	182.17
						Setup time (min)	30
						Take down time (min)	30
						Total time (min)	242.17

The workflow detailed in this work provides an automated methodology for TPS validation for the Halcyon ring gantry linear accelerator via a script that can easily be shared between centers and used common equipment found in most centers. This methodology allows the user to select the desired dose algorithms and calculation resolutions within the TPS. This is a general methodology for beam data validation that allows a user to select what they want to validate directly in the TPS and use the information to control the measurement via the generation of an XML file that will be run on the machine. The integrity of this file can be checked via a simple checksum^27^ to ensure it has not changed between generation in TPS to running on the machine. Once validated this provides confidence in the scan parameters settings and provides consistency in the validation by eliminating the manual setting of the scan parameters outside of the TPS typically seen during manual water tank measurements.

This methodology also allows the user to independently validate the beam data with any set of measurements they would like, using the equipment they like. The user can change the detectors, scan speeds, and phantom size used, as well. This improved flexibility could allow the user to test the beam data they feel are necessary using a relatively inexpensive TG‐51 compliant water tank and a data logging electrometer. This small form factor is very advantageous as all the equipment (tank, cables, detectors, electrometers, and platform) can be transported easily with two simple cases which can fit in the trunk of a car or be checked as luggage on a commercial flight. The custom‐made platform cost approximately $1,500 and was constructed in our campus machine shop. This adds convenience by allowing the user to quickly offset the tank reproducibly but is not required as one could still offset the tank without the platform. This system allows for significant flexibility for departments with multiple satellite locations that may be geographically separated. Instead of purchasing multiple 3D tanks or arranging transportation via moving truck from site to site, a user can easily transport a single 1D tank with their detectors of choice in an airtight molded plastic case.

Future work will include deploying this tool across multiple centers to determine the reproducibility of the system with both different treatment machines and different users deploying the system. Theoretically, the standardization and proper documentation of this method should allow any user to quickly validate their Halcyon and TPS with similar results. Ideally, this can be shown to be effective in a range of environments including academic centers, community practices, and resource limited environments. Potentially the design could be further optimized to improve the efficiency and ease of transportation.

## 5 CONCLUSION

This work has shown a robust and automated methodology for beam data validation for a preconfigured ring gantry linear accelerator. The TPS predicted dose shows excellent agreement with measurement with all data points being within 1.5% dose difference or 2 mm distance to agreement.

## 6 FUNDING AND CONFLICT OF INTEREST

The authors Dr. Nels Knutson, Dr. Geoffrey Hugo, Dr. Sasa Mutic, and Dr. Bin Cai receive research funding from Varian Medical Systems relating to this work and other works. The authors Dr. Nels Knutson, Mr. Matt Schmidt, Dr. Bin Cai, and Dr. Sasa Mutic receive income for consulting from Varian Medical Systems for work that is outside of this study. This work was completed with financial and nonfinancial support from Varian Medical Systems and Washington University in St. Louis Department of Radiation Oncology.
